# Bismuth Film-Coated Gold Ultramicroelectrode Array for Simultaneous Quantification of Pb(II) and Cd(II) by Square Wave Anodic Stripping Voltammetry

**DOI:** 10.3390/s21051811

**Published:** 2021-03-05

**Authors:** Sandra Enn D. Bahinting, Analiza P. Rollon, Sergi Garcia-Segura, Vince Carlo C. Garcia, Benny Marie B. Ensano, Ralf Ruffel M. Abarca, Jurng-Jae Yee, Mark Daniel G. de Luna

**Affiliations:** 1 Department of Chemical Engineering, University of the Philippines Diliman, Quezon City 1101, Philippines; sandraenn@gmail.com (S.E.D.B.); aprollon@up.edu.ph (A.P.R.); vincegarcia28@gmail.com (V.C.C.G.); 2 Environmental Engineering Program, National Graduate School of Engineering, University of the Philippines Diliman, Quezon City 1101, Philippines; ralfabarca@gmail.com; 3 Nanosystems Engineering Research Center for Nanotechnology-Enabled Water Treatment, School of Sustainable Engineering and the Built Environment, Arizona State University, Tempe, AZ 85287-3005, USA; sergio.garcia.segura@asu.edu; 4 University Core Research Center for Disaster-free and Safe Ocean City Construction, Dong-A University, Busan 49315, Korea; bmensano@dau.ac.kr; 5 Department of Architectural Engineering, Dong-A University, Busan 49315, Korea

**Keywords:** anodic stripping voltammetry, bismuth film electrode, electroanalysis, environmental water analyses, heavy metal detection, ultramicroelectrode array, water quality

## Abstract

The widespread presence of heavy metals in drinking water sources arises as a major health concern, particularly in developing countries. The development of low-cost and reliable detection techniques is identified as a societal need to provide affordable water quality control. Herein, a bismuth film-coated gold ultramicroelectrode array (BF-UMEA) was used for the detection of Pb(II) and Cd(II) in water samples via square wave anodic stripping voltammetry (SWASV). Experimental parameters such as deposition time, Bi(III) concentration, acetate buffer concentration, pH, square wave frequency, amplitude, and step potential were all varied to determine their effects on the current peak intensities of the target metal ions. Ten-fold excess in the concentration of interferences was found to cause a decrease in the stripping peak areas of Cd(II) and Pb(II) in the following order of magnitude: benzene < NaCl < Ni(II) < Cu(II). Using Box–Behnken design, the optimum SWASV parameters that provided maximum current peak areas were 14.76 Hz (frequency), 50.10 mV (amplitude), and 8.76 mV (step potential). The limits of detection of the as-prepared BF-UMEA were 5 and 7 µg L^−1^ for Pb(II) and Cd(II), respectively. These results demonstrate the potential use of a BF-UMEA in SWASV for the trace quantification of Pb(II) and Cd(II) in water samples.

## 1. Introduction

Heavy metal pollution in water bodies remains a major environmental concern. Although heavy metals are naturally occurring elements, their increased concentrations in aquatic environments are heavily linked to anthropogenic sources such as mining, metal plating, battery production, or their use as inorganic pigments [1]. Unfortunately, heavy metals do not degrade and tend to bioaccumulate throughout the food chain, thereby increasing the risks for hazardous health effects associated with metal toxicity and carcinogenicity [2]. Cadmium (Cd) and lead (Pb) are among the heavy metals included in the top 10 of the 2019 Substance Priority List by the United States Agency for Toxic Substances and Disease Registry. Numerous pieces of medical evidence proved that cadmium and lead exposure directly impairs the brain, lungs, bones, liver, and kidneys of a person [3,4]. These metals can even transfer to an embryo through the placenta, affecting fetal growth and development [5]. The World Health Organization (WHO) had set a maximum allowable concentration of 3 μg L^−1^ for Cd and 10 μg L^−1^ for Pb in drinking water [6,7].

Adequate water quality control is required to ensure the health and well-being of the human population. Developing countries rely mostly on private wells and groundwater as drinking water sources, which pose the highest risk for heavy metal contamination. Progress in water remediation technologies such as the use of adsorbents, ion exchange, and more recently, capacitive deionization has been realized [8,9]. However, the monitoring and measurement of heavy metals in actual environmental samples still rely on expensive spectroscopic analytical techniques such as electrothermal atomic absorption spectrometry (ET-AAS), flame atomic absorption spectrometry (FAAS), inductively coupled plasma mass spectrometry (ICP-MS), and inductively coupled plasma optical emission spectrometry (ICP-OES) [10]. Aside from bulky and high-maintenance instrumentation, spectroscopic measurements are time-consuming and require highly specialized personnel and tedious procedures for sample storage, handling, and preparation [11]. Thus, affordable and user-friendly analytical methods are continuously being developed to ensure good water quality in developing communities [12]. In this frame, electroanalytical sensing emerges as a competitive technology for water quality control with a broad niche market opportunity. 

Anodic stripping voltammetry (ASV) is an electroanalytical technique that can provide high sensitivity for trace metal analyses in a variety of environmental matrices [13]. Its exceptional sensitivity is attributed to the preconcentration step, wherein the metal accumulates onto the surface of the electrode by applying a sufficient negative potential through which the target analyte is reduced to form an amalgam with the electrode [14]. Following the preconcentration step, the analyte is oxidized back into the solution during an appropriate anodic scan, stripping and yielding a current response linear to the metal concentration [15]. This procedure enhances both the sensitivity and the selectivity towards the target analyte. 

Mercury-based electrodes have been traditionally used in ASV electroanalysis due to their wide negative potential range, high signal-to-noise ratio, low background current, and strong affinity to metals [16]. However, these types of electrodes are being replaced due to the highly toxic nature of mercury and more stringent regulations on its export and storage [17]. Alternative electrode materials for environmental applications and water quality sensing are thus being sought. In line with this, bismuth is drawing substantial attention as an attractive alternative to mercury in the field of electrochemical stripping analysis [18].

Bismuth film electrodes have lower toxicity while maintaining desirable electrochemical properties comparable to mercury electrodes, which include: (1) a wide negative potential window and (2) the ability to form fused alloys with other metal ions [15]. Bi-film electrodes are widely used in the determination of metals that have reduction potential more positive than Bi(III), such as thallium, antimony, zinc, copper, lead, and cadmium [19,20,21]. To prepare Bi-film electrodes, Bi(III) ions are usually electrodeposited on an electrode substrate via ex situ or in situ preparation. Although widely employed, ex situ plating is limited by the insufficient attachment of the bismuth film on the electrode surface, which greatly affects the sensor performance and lifespan. In an attempt to overcome this limitation, Hwang et al. [22] fabricated a Bi-film electrode by screen-printing the bismuth oxide on a glassy carbon electrode, then electrochemically reducing it to bismuth in an alkali solution. The as-prepared electrode featured a strong adherence between the bismuth film and the electrode surface, providing good stability and reproducibility up to ten repetitive measurements. In another study, NaBr salt was added into an acetate solution, yielding a denser and homogeneous growth of smaller Bi crystals on the electrode surface [23]. Meanwhile, in situ plating is still the more preferred method in the fabrication of Bi-film electrodes owing to its simplicity and shorter analysis time. In a comparative study of bismuth-modified screen-printed electrodes for lead detection, the in situ prepared electrode showed better analytical characteristics compared to ex situ and bulk plating method [24]. 

Different substrate materials have been explored as electrode support for Bi stripping voltammetry applications. Hwang et al. [25] used a carbon nanotube, glassy carbon, activated carbon and a graphite electrode coated with bismuth film for the simultaneous detection of lead, cadmium and zinc, and found out that the bismuth modified carbon nanotube electrode achieved the highest sensitivity to the trace metals. An inexpensive and disposable bismuth-coated copper mini-sensor was also investigated for the detection of trace lead and cadmium in river samples [26]. However, the large area of macroelectrodes typically used in Bi-film electrode fabrication presents an economic drawback since it requires a relatively higher amount of bismuth for coating. In addition, the mass transfer of the analyte from the bulk solution to the electrode surface is affected negatively due to the higher concentration of electron-changing species at the surface [27]. As such, the use of microelectrodes or ultramicroelectrodes (UME) for stripping analysis has been explored. The small dimensions of UME (i.e., typically less than or equal to the diffusion layer thickness) contribute to the enhancement of signal-to-noise ratios, a decrease in ohmic drop and the improvement of the mass transfer coefficient [28]. This allows UME to be used in highly resistive media and in very fast scan-rate voltammetric experiments [27]. Nonetheless, the weak current produced by an individual ultramicroelectrode remains a bottleneck for its analytical application [28]. Recently, the utilization of an ensemble of ultramicroelectrodes operating in parallel, also known as ultramicroelectrode array (UMEA), was proven to amplify the current and improve the sensitivity and the detection limit of the device. 

While several studies have focused on the analysis of heavy metals using mercury-coated UMEA [29], only a few explored the use of a bismuth film-coated ultramicroelectrode array. Additionally, based on an extensive literature review, no optimization studies have been performed yet via a statistical method on the square wave (SW) parameters for the quantification of heavy metal ions in water samples. Hence, in this study, an in situ prepared bismuth film-coated gold ultramicroelectrode array (BF-UMEA) was proposed as a sensing device for Cd(II) and Pb(II) detection in water samples by Square Wave Anodic Stripping Voltammetry (SWASV). The effects of varying parameters (i.e., deposition time, Bi(III) concentration, acetate buffer concentration, pH, square wave frequency, amplitude, and step potential) and interferences (i.e., Cu(II), Ni(II), NaCl and benzene) on current peak intensities of the target metal ions were evaluated. Finally, SWASV parameters including frequency, amplitude, and step potential were optimized using Box–Behnken design, and their interaction effects were studied. 

## 2. Materials and Methods

### 2.1. Materials and Chemicals

All reagents were analytical grade and used as received. Pb(NO_3_)_2_, Cd(NO_3_)_2_, and Bi(NO_3_)_3_·5H_2_O were purchased from Merck. Glacial acetic acid (CH_3_COOH) and anhydrous sodium acetate (C_2_H_3_NaO_2_), supplied by Theo-Pham Trading Corp. (Metro Manila, Philippines) and Instruchem, Inc. (Mandaluyong City, Philippines), respectively, were used to prepare the acetate buffer solution. Cu(NO_3_)_2_, Ni(NO_3_)_2_, NaCl, and benzene were obtained from Merck. All solutions were prepared using Millipore Milli-Q ultra-pure water with resistivity > 18.2 MΩ cm at 25 °C.

### 2.2. Electrochemical Device

**Figure 1:**
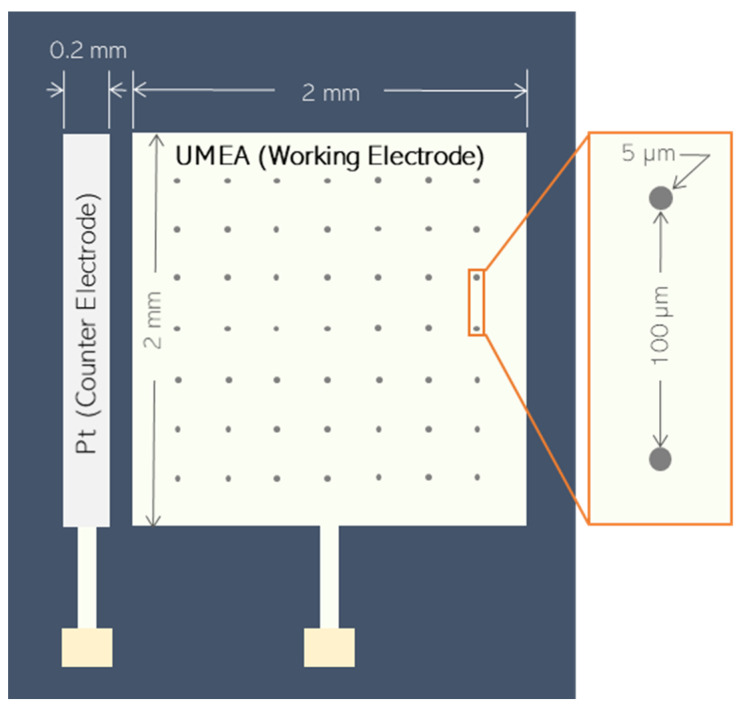
Design features of ultramicroelectrode arrays (UMEAs).

Electroanalysis was conducted in a three-electrode cell system. A gold ultramicroelectrode array (Au-UMEA) was used as the working electrode for in situ bismuth film deposition, while a leakless miniature Ag/AgCl electrode and platinum were employed as reference and external counter electrodes, respectively. The geometry and dimensions of the Au-UMEA are depicted in Figure 1 as adapted from a previous study [27]. The Au-UMEA (2 mm × 2 mm) contained an array of 400 gold microdiscs (5 μm diameter, 7.85 × 10^−5^ cm^2^ total area) at 100 μm apart. It is built with an on-chip counter electrode (Pt) which has a dimension of 0.2 mm × 2 mm, and it was placed 0.5 µm away from the working electrode.

### 2.3. In Situ Electrodeposition of Bismuth

Electrochemical activation of the gold ultramicroelectrode array was conducted by applying 15 scan cycles within the potential range of +0.2 V to −2.2 V (vs. Ag/AgCl reference electrodes) in a 0.1 M KNO_3_ solution, at a scan rate of 100 mV s^−1^. Activation of the UMEA was performed after every set of experiments to prevent any possible contamination effect from remaining residuals from the previous analysis. This step ensures that the electrode is in perfect condition even if stored for a short period. 

Bismuth coating was then prepared via in situ potentiostatic electrodeposition in a solution containing 10 mg L^−1^ of Bi(III) at a potential of −1.4 V. The SWASV conditions were as follows: 600 s deposition time (*t_dep_*), 15 Hz frequency (*f*), 50 mV pulse amplitude (*E_sw_*), 10 mV step potential (∆*E_s_*), 10 s rest time, and 0.8 V cleaning potential at 30 s cleaning time. Voltammetric measurements were performed using an EA160 EDAQ potentiostat connected to a computer system with Echem v1.6 EDAQ software.

### 2.4. Electroanalytical Methods

Electrochemical preconcentration time (180–900 s), Bi(III) concentration (300–1700 µg L^−1^), acetate buffer concentration (0–0.1 M), pH (3.2–4.0), square wave frequency (10–20 Hz), amplitude (20–80 mV), and step potential (5–15 mV) were all varied to examine their effects on the current peak intensity and square wave anodic stripping voltammograms of Cd(II) and Pb(II). With the exclusion of the parameter being studied, the solution contained 30 µg L^−1^ Pb(II), 50 µg L^−1^ Cd(II), 1 mg L^−1^ Bi(III) and 0.05 M acetate buffer at pH = 4.2 ± 0.1, and the SWASV conditions were: –1.20 V *E_dep_*, 600s *t_dep_*, 15 Hz *f*, 50 mV *E_sw_*, 10 mV ∆*E_s_*. Afterwards, the analytical performance of the BF-UMEA in terms of repeatability, stability and sensitivity was determined. The repeatability test of the BF-UMEA was carried out by consecutive measurements using a solution containing 20 µg L^−1^ Pb(II) and 40 µg L^−1^ Cd(II) concentrations without any activation of the BF-UMEA in between analyses. Meanwhile, the sensitivity and the limits of detection were determined using standard solutions of Pb and Cd ions at a calibration range of 1 to 10 µg L^−1^. The selectivity of the analytical method was further evaluated in the presence of interfering species (i.e., Cu(II), Ni(II), sodium chloride, and benzene) at concentrations of 100 and 500 µg L^−1^. Finally, Box–Behnken experimental design was utilized to further optimize the SWASV parameters (i.e., square wave frequency, step potential, and amplitude). The two-parameter interaction effects were also investigated, and the validity of the design was carried out by comparing the result of the actual and predicted values.

## 3. Results and Discussion

### 3.1. Effect of Experimental Variables

#### 3.1.1. Preconcentration Time

**Figure 2:**
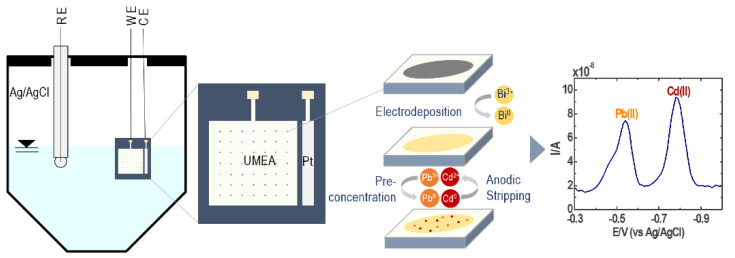
Mechanism of simultaneous quantification of Pb(II) and Cd(II) by square wave anodic stripping voltammetry (SWASV) using a bismuth film-coated gold ultramicroelectrode array (BF-UMEA).

The BF-UMEA detection of Pb(II) and Cd(II) via SWASV starts with the preconcentration step (Figure 2). By applying an appropriate deposition potential (−1.2 V) [22], bismuth film, which is electrodeposited on the surface of the Au-UMEA (Equation (1)), forms a fused alloy with the reduced lead and cadmium ions (Equation (2); M(II) = Pb(II), Cd(II)) [30]. Thereafter, the stripping step oxidizes back the metals to Pb(II) and Cd(II) upon anodic scan and diffuses out of the bismuth film into the solution, generating a current response linear to the metal concentration (Equation (3)).

(1) Bi(III)+Au-UMEA+3e→BF-UMEA

(2) M(II)+BF-UMEA+2e→M/Bi-UMEA

(3) M/Bi-UMEA→M(II)+BF-UMEA+2e

**Figure 3:**
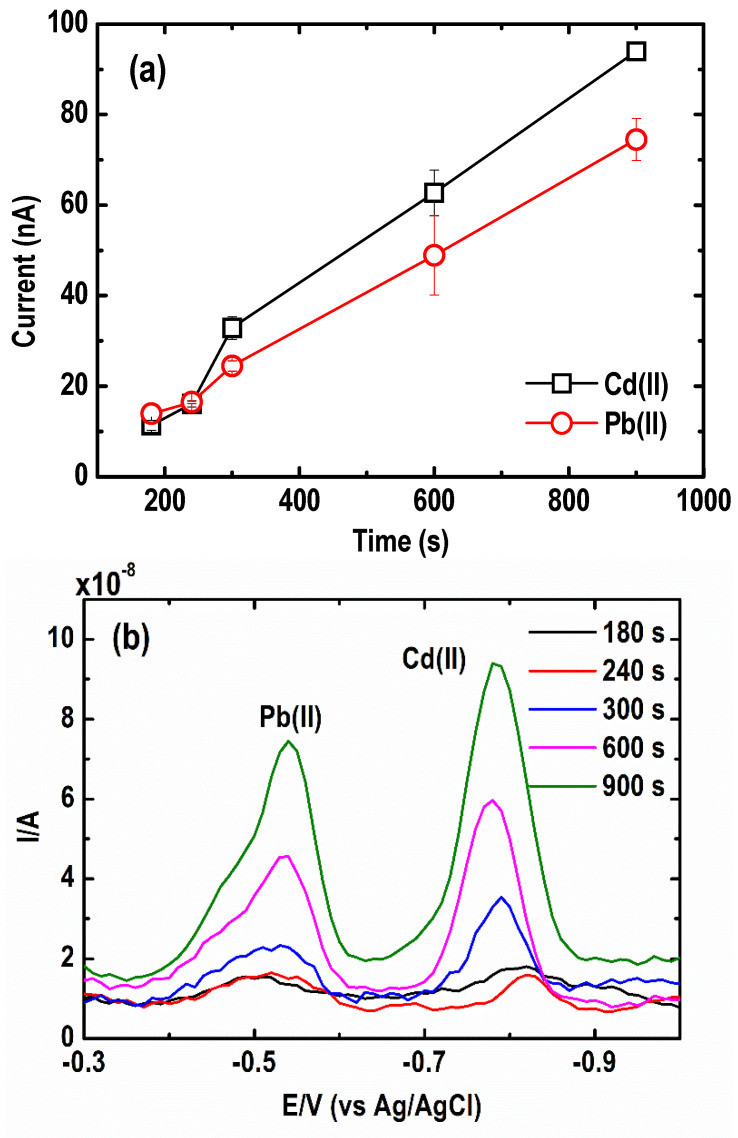
Effect of preconcentration time on (**a**) current peak intensity, and (**b**) square wave anodic stripping voltammograms of Pb and Cd (Conditions: [Pb(II)] = 30 µg L^−1^; [Cd(II)] = 50 µg L^−1^; [Bi(III)] = 1 mg L^−1^; [acetate buffer] = 0.05 M at pH = 4.2 ± 0.1; deposition potential = −1.20 V; SW frequency = 15 Hz; SW amplitude = 50 mV; SW step potential = 10 mV).

In this regard, preconcentration or deposition time plays a vital role in achieving higher sensitivity to trace concentrations of Pb(II) and Cd(II) [31]. Low detection limits by ASV, up to parts per billion, are reached mainly due to longer deposition times. Herein, the effect of the preconcentration time on the stripping peaks of Pb and Cd was determined in the range of 180 to 900 s using a Au ultramicroelectrode array as the substrate for Bi film electrodes. The solution contained 30 µg L^−1^ Pb(II) and 50 µg L^−1^ Cd(II) along with 1 mg L^−1^ Bi(III) in 0.05 M acetate buffer (pH = 4.2 ± 0.1). A higher concentration of Cd(II) with respect to Pb(II) was applied because Cd(II) has lower diffusivity or lower affinity for Bi than Pb(II) as a result of the parasitic hydrogen evolution reaction [32,33,34]. The peak current of the metals increases almost linearly up to a deposition time of 900 s, as shown in Figure 3a. In previous studies, longer deposition times caused electrode saturation by metal ions, especially in a solution containing higher metal concentrations [35]. However, such saturation, which causes a deviation from the linearity of the peak current with longer deposition time, was not observed in the present study. Therefore, 600 s was ultimately selected for the succeeding experiments as a compromise between relatively short analysis time and high sensitivity. This approach was beneficial for both Cd(II) and Pb(II) signals, as shown in the SWASV curves (Figure 3b).

#### 3.1.2. Bi(III) Concentration

The concentration of Bi(III) ions used in the in situ preparation of the BF-UMEA controls the thickness of the Bi film, which has been known to influence the height of the current peak of the target metals present in the solution [36,37]. Similar to the effect of Hg film thickness, thicker films present limitations on the mass transfer of metal ions diffusing out during the stripping step, while a saturation effect takes place in thin films due to the diffusion and excessive deposition of metal ions into the film. Therefore, an ideal film thickness for target analyte concentration should be determined by optimizing Bi(III) ion concentration. 

**Figure 4:**
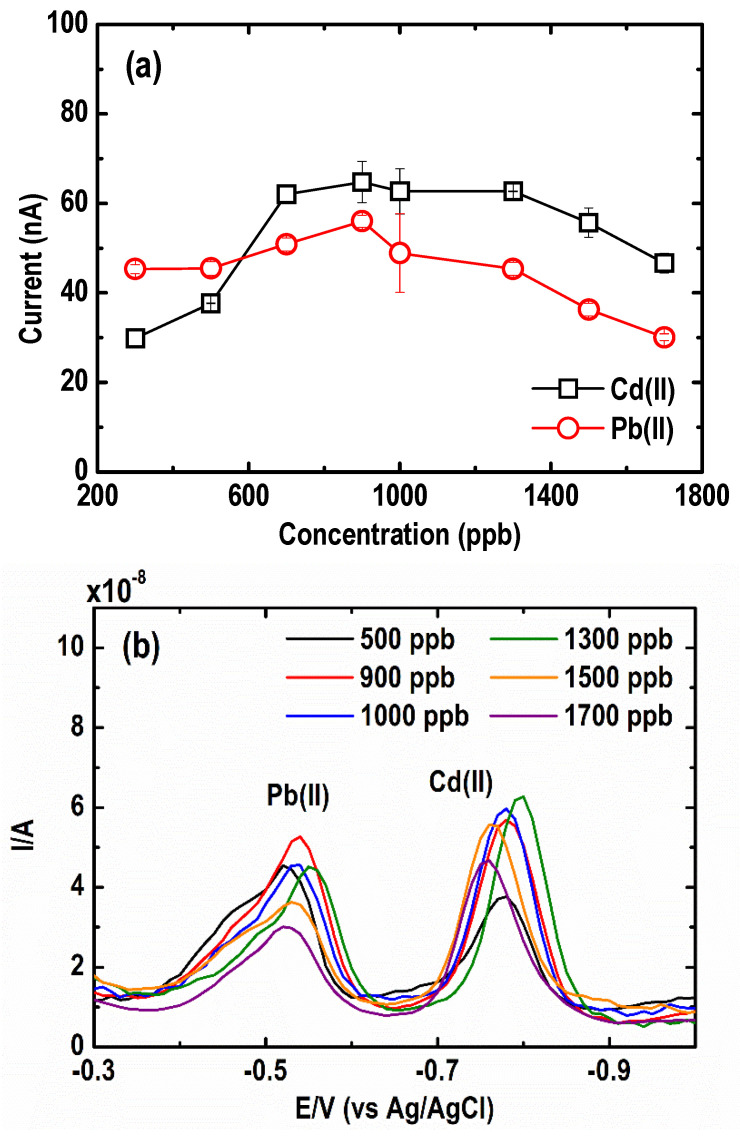
Effect of Bi(III) concentrations on (**a**) current peak intensity, and (**b**) square wave anodic stripping voltammograms of Cd and Pb. (Conditions: [Pb(II)] = 30 µg L^−1^; [Cd(II)] = 50 µg L^−1^; [acetate buffer] = 0.05 M at pH = 4.2 ± 0.1; deposition potential = −1.20 V; deposition time = 600 s; SW frequency = 15 Hz; SW amplitude = 50 mV; SW step potential = 10 mV).

The effect of Bi(III) ions on the magnitude and shape of the stripping peaks for 30 µg L^−1^ Pb(II) and 50 µg L^−1^ Cd(II) in 0.05 M acetate buffer solution at pH 4.2 ± 0.1 was investigated. The peak current intensity of both metals, as shown in Figure 4a, was recorded at different Bi(III) concentrations (300 to 1700 µg L^−1^). An increasing trend, for both Cd(II) and Pb(II) stripping peak heights, was observed when Bi(III) concentration was raised from 300 to 900 µg L^−1^, which was likely due to an increase in the nucleation site and alloy formation of metal and Bi(III) ions in the solution. Beyond 900 µg L^−1^ Bi(III) concentration, a reduction in both Cd(II) and Pb(II) peak currents was observed. This may be attributed to a possible saturation of Bi(III) ions on the BF- UMEA surface, resulting in a thick Bi layer that partially blocked the conductive surface of the electrode [35]. 

The SWASV curves at varying Bi(III) concentrations are illustrated in Figure 4b. As shown, the thickness of the film did not drastically affect the potential of the characteristic stripping peak position of the two metals [30]. Additionally, the peaks of Cd(II) and especially Pb(II) were asymmetrical and deformed. This has been similarly reported in other Bi-coated Au electrode studies which were ascribed to the formation of different types of either Cd or Pb surface alloys with the different kinds of Bi deposits [32,33]. A hump formed in the Pb stripping peak indicates the presence of two types of deposited Pb, one that formed an alloy with Bi and another that weakly interacted with it.

#### 3.1.3. Bi(III)-to-Metal Ion Ratio

**Figure 5:**
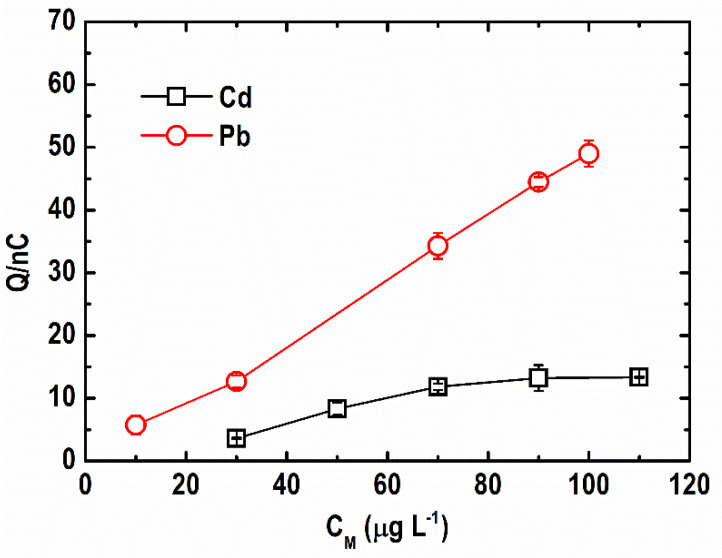
Effect of Bi(III)-to-metal ion ratio on stripping peak area of Cd and Pb. (Conditions: [Pb(II)] = [Cd(II)] = 10–130 µg L^−1^; [Bi(III)] = 500 µg L^−1^; [acetate buffer] = 0.05 M at pH = 4.2 ± 0.1; deposition potential = −1.20 V; deposition time = 600 s; SW frequency = 15 Hz; SW amplitude = 50 mV; SW step potential = 10 mV).

The effect of the Bi(III)-to-metal ion ratio on the ASV response of Cd and Pb ions was investigated in previous studies. It was reported that a Bi(III)-to-metal ion ratio of equal to or less than 10 is either optimal or adequate to attain metal stripping response [32,33]. The change in the stripping peak area for Pb(II) and Cd(II) at 10 to 130 µg L^−1^ concentrations in 500 µg L^−1^ Bi(III) ions is shown in Figure 5. Depending on the metal ion concentration, the level of Bi(III) excess for Pb(II) and Cd(II) was 5 to 50 fold and 4 to 17 fold, respectively. As shown, a lower than ten-fold excess of Bi(III) concentration is already sufficient to obtain higher stripping responses. Furthermore, the response of Pb(II) was stronger than Cd(II) even at low Bi(III) excess levels, signifying higher sensitivity and better Bi utilization for Pb(II) sensing.

#### 3.1.4. Acetate Buffer Concentration

**Figure 6:**
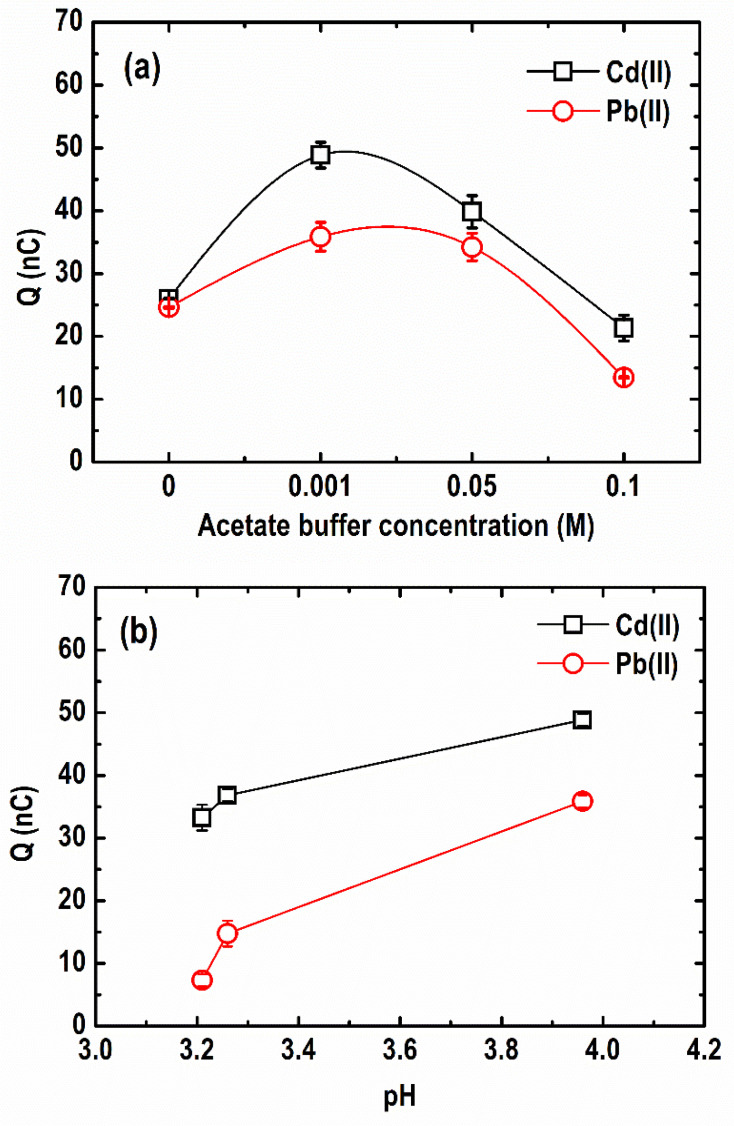
Comparison of Cd and Pb stripping peaks at different (**a**) acetate buffer concentrations and (**b**) pH (Conditions: [Pb(II)] = 30 µg L^−1^; [Cd(II)] = 50 µg L^−1^; [Bi(III)] = 1 mg L^−1^; deposition potential = −1.20 V; deposition time = 600 s; SW frequency = 15 Hz; SW amplitude = 50 mV; SW step potential = 10 mV).

A quantitative depiction of the difference in the stripping peaks of Cd(II) and Pb(II) at varying acetate buffer concentrations (0–0.1 M), each containing 30 µg L^−1^ Pb, 50 µg L^−1^ Cd(II), and 1 mg L^−1^ Bi(III), is illustrated in Figure 6a. As shown, a stripping response for both metals was obtained even in an unbuffered solution (0 M acetate; pH 3.2). Similar studies reported that it is possible to use unbuffered media using microelectrodes; however, the background current at more negative potentials is much higher in this case due to the high hydrogen evolution rates in more acidic solutions [32]. When the acetate buffer concentration increased from 0 to 0.001 M (pH 4.0), the stripping charge increased drastically from 26.03 and 24.65 nC to 48.87 and 35.86 nC for Cd(II) and Pb(II), respectively. Afterwards, it began to decline as the concentration of acetate buffer continued to rise from 0.001 M to 0.1 M (pH 4.4). This suggests that the formation of Pb-Bi and Cd-Bi alloys is strongly affected by the electrolyte concentration, which also dictates the ionic strength of the solution [38]. A similar trend was reported in previous studies [17,38,39,40]. Herein, the most sensitive responses to Cd and Pb were obtained in 0.001 M acetate buffer concentration. 

#### 3.1.5. pH

Figure 6b presents the variation in pH at optimum acetate buffer concentration (0.001 M) and its effect on the stripping charge of the metals. As shown, the stripping peaks of both Cd(II) and Pb(II) increased with an increment of pH from 3.2 to 4.0. The smaller response of the BF-UMEA at lower pH can be attributed to the higher proton (H^+^) concentration in the solution which competes against the target metals for the ion-exchange sites on the surface of the BF-UMEA, affecting the electrodeposition of the metal ions [41]. As the pH increases, deprotonation occurs, allowing surface complexation and electrostatic attraction between the metal ions and the electrode surface [42]. Hence, from the studied pH range, pH 4.0 provided the maximum stripping response for both Cd(II) and Pb(II) using the BF-UMEA. On the other hand, a further increase in the electrolyte pH may promote metal hydrolysis, which inhibits metal accumulation on the electrode surface [42]. For instance, Bi(III) ions easily hydrolyze in neutral and weakly basic electrolyte solutions and form white precipitates according to Equations (4) and (5) [43].

(4) $${\mathrm{Bi}}^{3+}+H_{2}O\to {\mathrm{BiO}}^{+}+2H^{+}$$

(5) $${\mathrm{Bi}}^{3+}+3H_{2}O\to \mathrm{Bi}{\left(\mathrm{OH}\right)}_{3}+3H^{+}$$

These hydroxy-complexes have less positive standard potential and are more difficult to electrochemically deposit on the Au-UMEA surface [32]. Such information can be useful in the simultaneous determination of Cd and Pb not just in wastewater, but also in other contaminated samples such as soil, blood and dairy products, where acid pre-treatment is required for analysis [40,44,45]. 

### 3.2. Effect of SWASV Parameters

#### 3.2.1. Square Wave Frequency 

**Figure 7:**
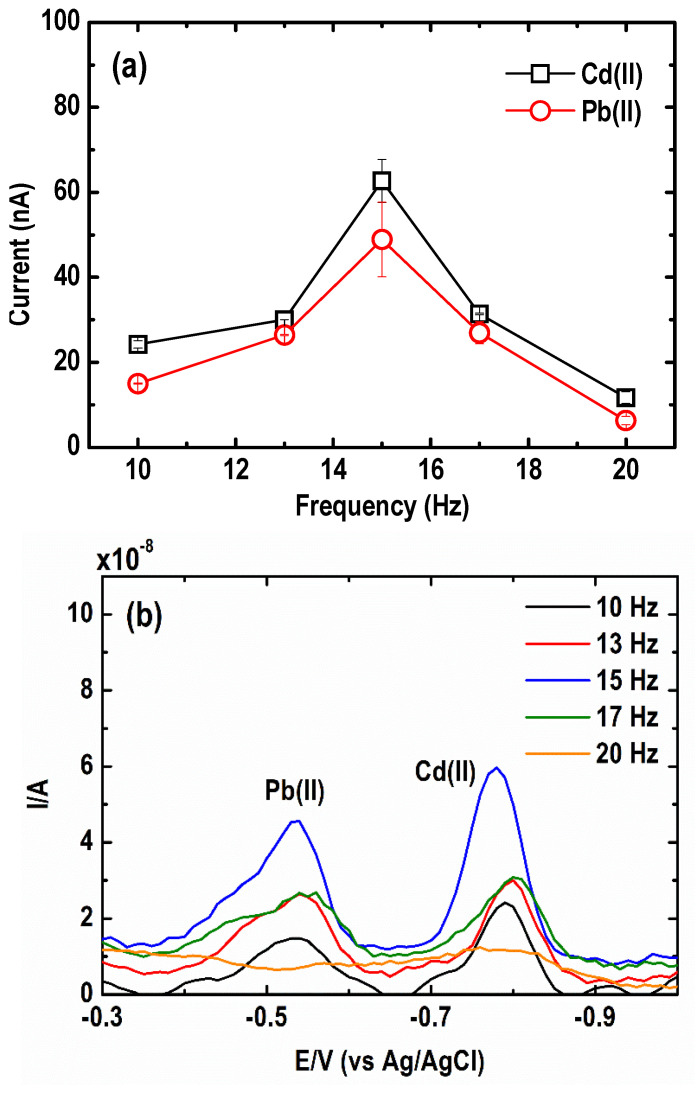
Effect of square wave frequency on the (**a**) current peak intensity and (**b**) square wave anodic stripping voltammograms of Pb and Cd. (Conditions: [Pb(II)] = 30 µg L^−1^; [Cd(II)] = 50 µg L^−1^; [Bi(III)] = 1 mg L^−1^; [acetate buffer] = 0.05 M at pH = 4.2 ± 0.1; deposition potential = −1.20 V; deposition time = 600 s; SW amplitude = 50 mV; SW step potential = 10 mV).

The effect of square wave frequency on the current signal of the Cd(II) and Pb(II) is presented in Figure 7a. At first, the peak signal increased dramatically when the frequency was raised from 10 to 15 Hz, then it decreased relatively fast as the frequency went up to 20 Hz. According to Li et al. [41], both the square wave frequency and the step potential define an effective scan rate. At SW frequency greater than 15 Hz, a faster scan rate was observed, which is not ideal since it can result in the broadening of the stripping peaks and weak discrimination against the steep sloping baseline as a result of the higher background current [46]. These explain why the voltammograms of Pb(II) and Cd(II) in Figure 7b show a lower peak resolution, worse peak baseline, and peak distortion at a square wave frequency of 20 Hz. The optimum frequency ranged from 13 to 17 Hz, in which an improvement in both peak resolution and the peak current was therefore obtained.

#### 3.2.2. Square Wave Amplitude 

**Figure 8:**
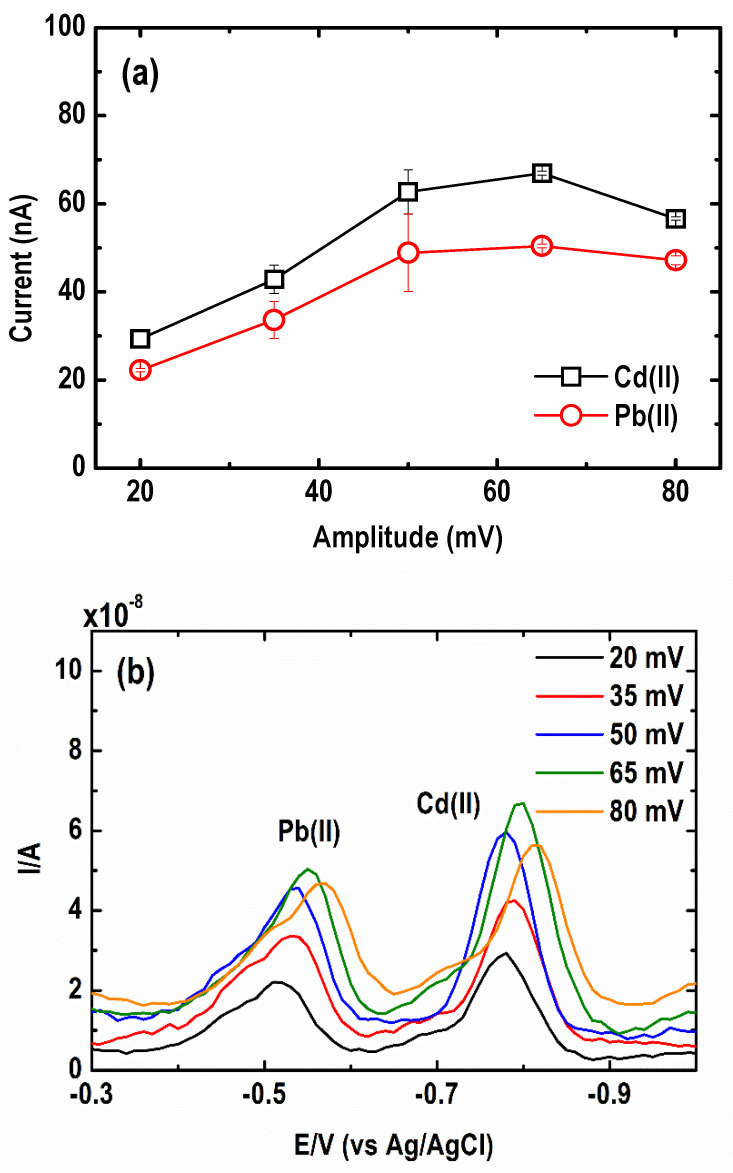
Effect of square wave amplitude on the (**a**) current peak intensity and (**b**) square wave anodic stripping voltammograms of Pb and Cd. (Conditions: [Pb(II)] = 30 µg L^−1^; [Cd(II)] = 50 µg L^−1^; [Bi(III)] = 1 mg L^−1^; [acetate buffer] = 0.05 M at pH = 4.2 ± 0.1; deposition potential = −1.20 V; deposition time = 600 s; SW frequency = 15 Hz; SW step potential = 10 mV).

The effect of varying amplitude from 20 to 80 mV on the SWASV current signal of the metal was also examined. As shown in Figure 8a, an increase in the amplitude from 20 to 65 mV led to a rise in the stripping net peak of Cd(II) and Pb(II). Beyond 65 mV, the current peak of both metal ions started to decrease. Meanwhile, Figure 8b shows that upon increasing the amplitude, the baseline of the voltammograms shifted towards higher currents, producing larger and broader peaks. The optimum amplitude ranged from 35 to 65 mV.

#### 3.2.3. Square Wave Step Potential 

**Figure 9:**
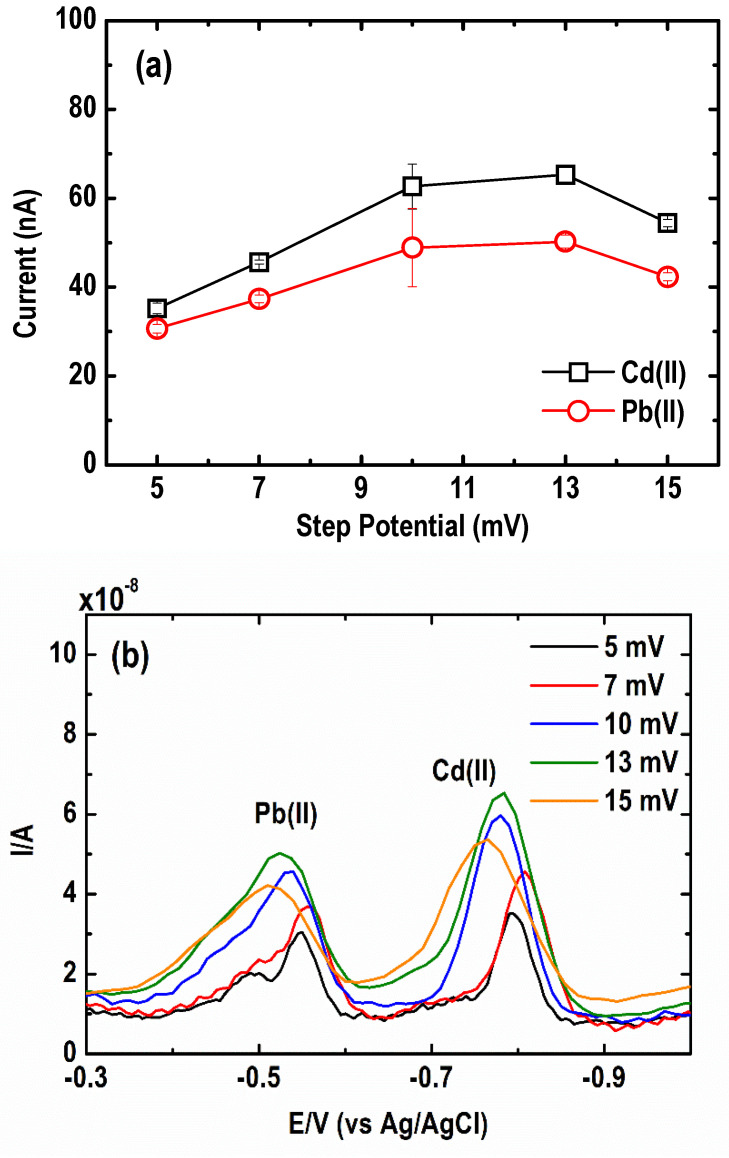
Effect of square wave step potentials on the (**a**) current peak intensity and (**b**) square wave anodic stripping voltammograms of Pb and Cd. (Conditions: [Pb(II)] = 30 µg L^−1^; [Cd(II)] = 50 µg L^−1^; [Bi(III)] = 1 mg L^−1^; [acetate buffer] = 0.05 M at pH = 4.2 ± 0.1; deposition potential = −1.20 V; deposition time = 600 s; SW frequency = 15 Hz; SW amplitude = 50 mV).

The effect of step potential on the SWASV current signal of the metal was examined by varying the SW step potential from 5 to 15 mV. As depicted in Figure 9a, an increasing current peak response was found in the step potential range of 5 to 13 mV. The square wave anodic voltammogram in Figure 9b shows a slight shift in the peak potentials towards positive values upon an increase in step potential. The optimum step potential is within the range of 7 to 13 mV.

### 3.3. Interference Study

Since water samples typically contain impurities other than the target metal ions, the effect of the different types of interferences such as metal cations (Cu(II) and Ni(II)), salt (NaCl), and an organic compound (benzene) on the stripping measurements of Pb and Cd was investigated in 0.05 M acetate buffer containing 30 µg L^−1^ Pb(II) and 50 µg L^−1^ Cd(II) along with 1 mg L^−1^ of Bi(III) ions (Table 1). A total disappearance of Cd and Pb electrochemical responses was observed in the presence of 100 and 500 μg L^−1^ copper ions (Table 1). According to previous studies, Cu(II) greatly influences the peak area response for both Cd and Pb ions at bismuth electrodes because Cu(II), having a reduction potential almost similar to that of bismuth, tends to compete with bismuth ions for the active sites on the electrode surface [17]. In addition, Cu(II) forms a metallic alloy with Pb and Cd ions [47] as well as Bi ions [48] during the deposition step. Similar results were also reported in a previous ASV study employing an oxygen plasma/bismuth modified inkjet printed graphene electrode [11]. Meanwhile, Ni(II), at 100 and 500 µg L^−1^ concentrations, remarkably suppressed the stripping response by 53.38% and 80.05% for Cd(II) and 9.99% and 49.72% for Pb(II), respectively. It is evident that Cd(II) is more susceptible to interferences from Ni(II) than Pb(II). However, both target analytes were already quantifiable in the presence of this interference. The addition of NaCl also greatly influenced the stripping response of both metals, causing a decline in peak current intensities by 34.27% and 43.59% for Cd(II) and Pb(II), respectively, when NaCl concentration was ~10-fold excess. The decrease in stripping response is ascribed to the inactivation of the bismuth surface by Cl^-^ ions due to the formation of BiCl_3_, or to the chloride complexation of Pb. Lastly, the presence of benzene only slightly suppressed the stripping response of the studied trace metals. However, at increasing benzene concentration, the interference on analytical responses of both metals likewise increased. The peak area reduction for Cd(II) improved from 26.58% to 29.83%, while for Pb(II) it improved from 10.87% to 14.29% when benzene concentration was raised from 100 to 500 µg L^−1^. The same phenomenon was observed at increasing amounts of Ni(II) and NaCl. In general, the effect of the interfering ions on the stripping signals of Cd(II) and Pb(II) increased in the following order: benzene < NaCl < Ni(II) < Cu(II).

The interference of cations on Pb and Cd stripping peaks could be effectively reduced without affecting the target metal ions by adding a masking agent in the sample solution prior to the heavy metal measurement. One example is the utilization of ferricyanide or ferrocyanide, which forms insoluble and stable complexes with copper, hence preventing the copper from competing with bismuth on the electrode active sites [49,50]. Moreover, to improve the selectivity of the sensor against anion interferences, a permselective film, such as Nafion, can be coated on the surface of the bismuth electrode. Wang et al. [51] reported that the cation-exchange ability of Nafion prevents both anions and surfactants from moving towards the working electrode, therefore decreasing their interferences to the sensor.

### 3.4. Optimization of the SWASV Parameters

Response surface methodology (RSM) is a powerful statistical tool used for designing experimental conditions and the optimization of process variables [52]. In this study, RSM based on Box–Behnken design (BBD) was used to optimize the SWASV parameters and to evaluate the interaction of these parameters and their effects on the current peak area/charge of Cd(II) and Pb(II) ions. The optimum range chosen for each parameter was based on the current peak area (high sensitivity) and peak resolution of the metal ions, as obtained from the results of the parametric studies. The three-factor, three-level BBD used for optimization comprised the following parameters: amplitude (35, 50, and 65 mV), frequency (13, 15, and 17 Hz), and step potential (7, 10, and 13 mV). The data obtained from performing the sets of runs generated by the optimum range for each parameter were fitted to an empirical model. After this, the correlation of the current peak area for Cd(II) and Pb(II) ions with the three parameters was created. In terms of dimensionless coded values, the quadratic equation for the peak current area is given by Equations (6) and (7), while the model in terms of actual factors is shown in Equations (8) and (9) for Cd(II) and Pb(II), respectively.

Peak Current Area, Cd (nC) = +26.92 − 11.64 A + 2.29 B − 2.75 C + 1.48 AB − 1.50 AC − 0.66 BC − 2.750 × 10^−3^ A^2^ + 6.37 B^2^ + 5.42 C^2^ (6)

Peak Current Area, Pb (nC) = +22.38 − 3.35 A + 0.94 B − 0.54 C + 1.99 AB − 1.97 AC + 2.37 BC − 4.54 A^2^ − 0.90 B^2^ − 1.50 C^2^ (7)

where A, B and C refer to SW frequency, SW amplitude, and SW step potential, respectively.

Peak Current Area, Cd (nC) = +238.74 − 5.76 F − 3.27 A − 8.47 SI + 0.049 FA − 0.25 FSI − 0.014 ASI − 6.88 × 10^−4^ F^2^ + 0.028 A^2^ + 0.60 SI^2^ (8)

Peak Current Area, Pb (nC) = −208.99 + 32.33 F − 1.06 A + 5.44 SI + 0.066 FA − 0.33 FSI + 0.053 ASI − 1.13 F^2^ − 4.01 × 10^−3^ A^2^ − 0.17 SI^2^ (9)

where A is the amplitude (mV), F is the frequency (Hz), and SI is the step potential (mV).

In the dimensionless model for Cd(II), factor A, which corresponds to the frequency, has the largest negative coefficient, indicating that it has the most significant negative impact on the current peak area. The step potential also negatively affects the peak current area, albeit to a lesser degree compared to frequency. Meanwhile, the amplitude has a positive effect on the current peak area. Lastly, the interaction effect AB has positive effects on the current peak area opposite to BC and AC. The model for Pb(II) shows the same effect from the factors.

**Figure 10:**
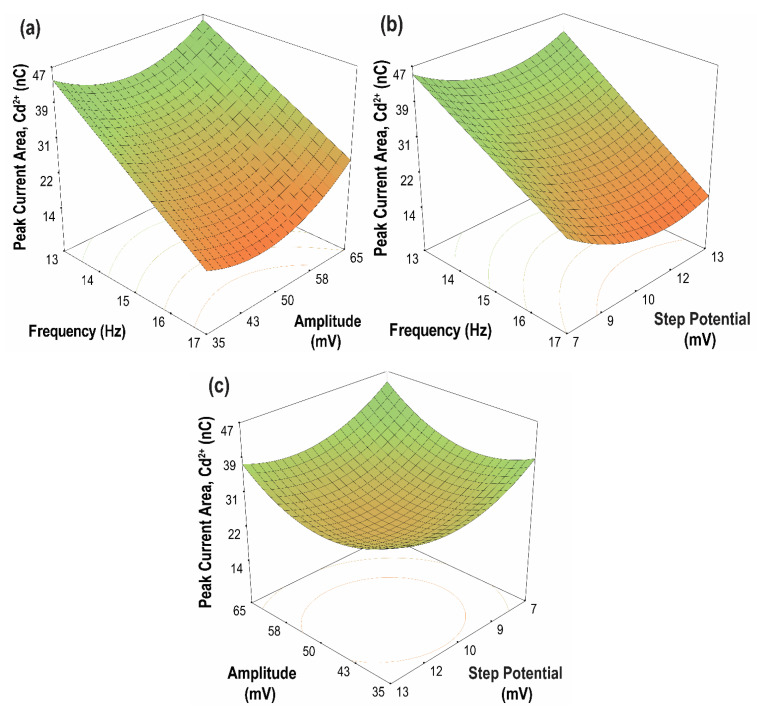
Contour plots of the two parameter interaction effects on the peak current area of Cd(II) (**a**) frequency vs. amplitude, (step potential = 10 mV); (**b**) frequency vs. step potential, (amplitude = 50 mV); (**c**) amplitude vs. step potential, (frequency = 15 Hz).

**Figure 11:**
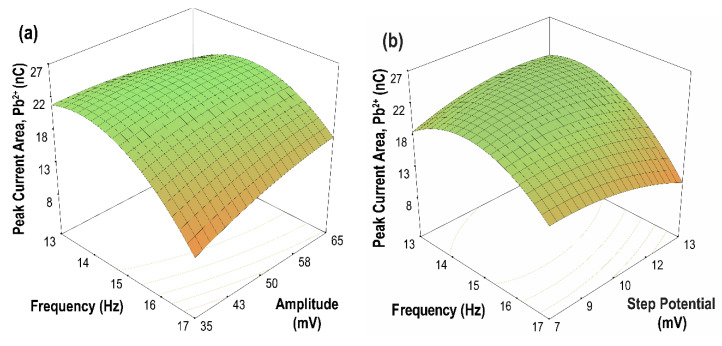

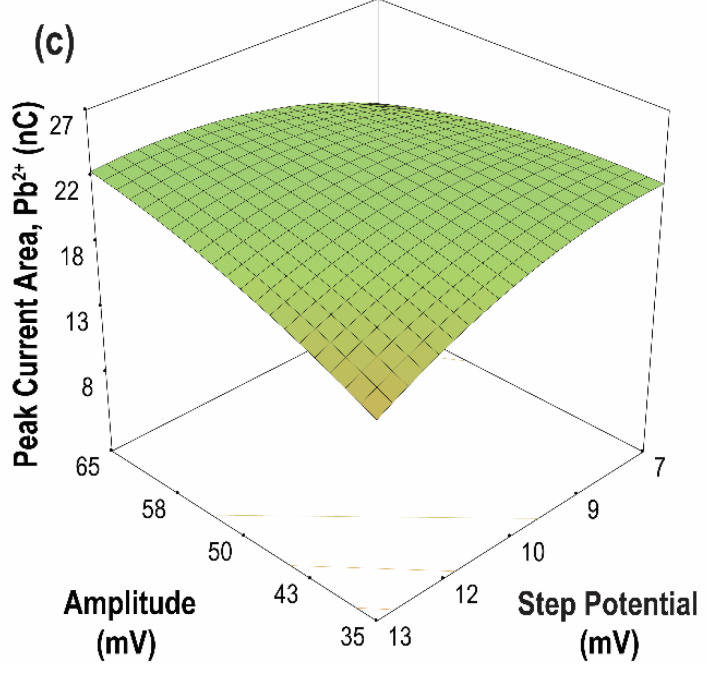
Contour plots of the two parameter interaction effects on the peak current area of Pb(II) (**a**) frequency vs. amplitude, (step potential = 10 mV); (**b**) frequency vs. step potential, (amplitude = 50 mV); (**c**) amplitude vs. step potential, (frequency = 15 Hz).

The contour plots of the two-parameter interaction effects for the current peak area of Cd(II) and Pb(II) are shown in Figure 10 and Figure 11, respectively. The highest current peak area of Cd(II) and Pb(II) was achieved for an increasing amplitude at a lower frequency. Moreover, for parameter AC, low values of both frequency and step potential yield a high current peak area for both Cd(II) and Pb(II). Meanwhile, for parameter BC, a high current peak area for Cd(II) was achieved by increasing the amplitude and decreasing the step potential. In contrast, a high current peak was obtained for Pb(II) when both amplitude and step potential were increased. In general, high sensitivity can be achieved at high amplitude and low frequency and step potential.

The optimal conditions that provided the maximum current peak areas are as follows: 14.76 Hz (SW frequency), 50.10 mV (SW amplitude), and 8.76 mV (SW step potential). These optimized values were fitted to the empirical model, which gave the predicted current peak areas of 30.35 nC and 22.59 nC for Cd(II) and Pb(II), respectively. Verification experiments were then conducted, and the actual current peak areas obtained were 31.39 nC for Cd(II) and 23.28 nC for Pb(II). The proximity between predicted and experimental values for both metal ions verifies the suitability of the model for the investigated parameters.

### 3.5. Analytical Performance of BF-UMEA

After all the parameters have been investigated, the following optimum values (i.e., deposition potential of −1.2 V; deposition time of 600 s; Bi(III) ion concentration of 900 µg L^−1^; 0.001 M acetate buffer (pH = 4 ± 0.1); SW frequency of 14.76 Hz; SW amplitude of 50.10 mV; and SW step potential of 8.76 mV) were used in the SWASV experiments to determine the analytical performance of the BF-UMEA in terms of repeatability, stability and sensitivity. The repeatability of the BF-UMEA was evaluated by taking five repetitive SWASV measurements of combined 20 µg L^−1^ Pb(II) and 40 µg L^−1^ of Cd(II) in 0.001 M acetate buffer solution (pH = 4 ± 0.1). The relative standard deviations (RSDs) obtained were 5.88% for Pb(II) and 8.75% for Cd(II). These results are comparable with other studies employing bismuth-based sensors for the detection of Cd(II) and Pb(II) in water matrices [53,54]. The stability of the BF-UMEA was assessed after 11 days of storage at 25 °C and the RSDs were 4.53% and 5.17% for Pb(II) and Cd(II), respectively, signifying the long-term stability of the sensor [55].

**Figure 12:**
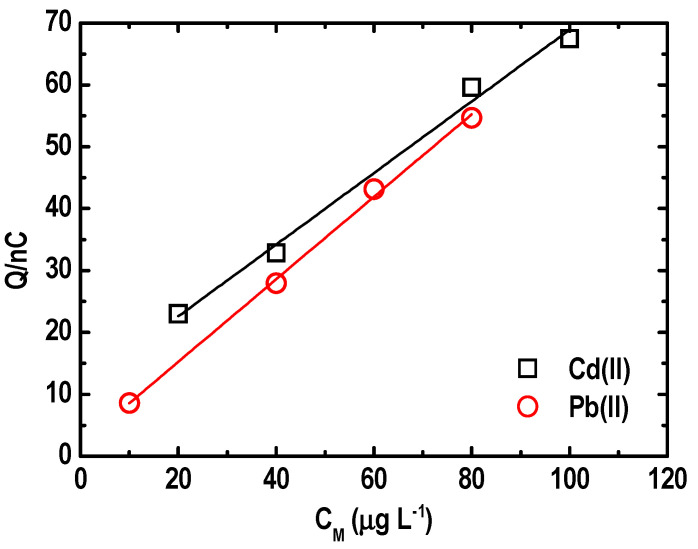
Calibration curve for an increasing concentration of Pb(II) and Cd(II) ions on an in situ BF-UMEA by SWASV.

Meanwhile, calibration was performed on the BF-UMEA for the simultaneous determination of target metal ions in a concentration range of 10 to 80 µg L^−1^ for Pb(II) and 20 to 100 µg L^−1^ for Cd(II) (Figure 12). A linear relationship was obtained between the magnitude of the stripping charge and the concentration of each metal ion, with correlation coefficient (*R*^2^) values equal to 0.9897 for Cd(II) and 0.9971 for Pb(II). Data fitted equations are given in Equation (10) and Equation (11) for Cd(II) and Pb(II), respectively:

(10) QnC=11.0+0.579[Cd(II)/μg L−1]

(11) QnC=1.91+0.667 [Pb(II)/μg L−1]

The slope of the linear fit (sensitivity) was 0.58 nC L µg^−1^ for Cd(II) and 0.67 nC L µg^−1^ for Pb. The limits of detection (LOD) were determined to be 5 and 7 μg L^−1^ for Pb(II) and Cd(II), respectively. This suggests that the BF-UMEA outperformed other bismuth-based electrodes in the simultaneous quantification of trace Pb(II) and Cd(II) from water matrices [26,47,56]. 

## 4. Conclusions

This present work demonstrates the performance of a BF-UMEA for the simultaneous determination of Cd(II) and Pb(II) in water by SWASV. Optimal experimental conditions of 600 s and 900 µg L^−1^ were determined for deposition time and Bi(III) concentration, respectively. Moreover, low acetate buffer concentrations at pH less than the *pKa* of acetic acid resulted in a high response in the current peak area for both Cd(II) and Pb(II). The results also show that Ni(II), Cu(II), NaCl, and benzene interferences caused significant reductions in the peak area responses for both analytes. Instrument conditions were optimized, and the values were validated by Box–Behnken design. The set up obtained low detection limits of 7 and 5 µg L^−1^ for Cd(II) and Pb(II), respectively, with a relative standard deviation of 10.14% for Cd(II) and 11.18% for Pb(II). The results indicate that the use of a BF-UMEA in SWASV for the detection of Cd(II) and Pb(II) in water is a promising alternative to existing analytical methods with its low detection limits and simple setup.

## Figures and Tables

**Table 1:** Effects of addition of potential interferences on the peak area of Pb(II) and Cd(II).

	Concentration	Peak Area Reduction (%)	
Interference	(μg L^−1^)	Cd(II)	Pb(II)
Cu(II)	100	No peak	No peak
	500	No peak	No peak
Ni(II)	100	53.38	9.99
	500	80.05	49.72
NaCl	100	7.94	21.11
	500	34.27	43.59
Benzene	100	26.58	10.87
	500	29.83	14.29

## Data Availability

Data are contained within the article.
